# Integrated analysis of transcriptomic and metabolomic profiling reveal the p53 associated pathways underlying the response to ionizing radiation in HBE cells

**DOI:** 10.1186/s13578-020-00417-z

**Published:** 2020-04-15

**Authors:** Ruixue Huang, Xiaodan Liu, He Li, Yao Zhou, Ping-Kun Zhou

**Affiliations:** 1 Department of Occupational and Environmental Health, Xiangya School of Public Health, Central South University, Changsha, Hunan 410078 China; 2 Department of Radiation Biology, Beijing Key Laboratory for Radiobiology, Beijing Institute of Radiation Medicine, AMMS, Beijing, 100850 China; 3 Institute for Chemical Carcinogenesis, State Key Laboratory of Respiratory, School of Public Health, Guangzhou Medical University, Guangzhou, 511436 People’s Republic of China

## Abstract

**Background:**

Radiation damage to normal tissues is a serious concern. P53 is a well-known transcription factor which is closely associated with radiation-induced cell damage. Increasing evidence has indicated that regulation of metabolism by p53 represents a reviving mechanism vital to protect cell survival. We aimed to explore the interactions of radiation-induced transcripts with the cellular metabolism regulated by p53.

**Methods:**

Human bronchial epithelial (HBE) cell line was used to knockout p53 using CRISPR/cas9. Transcriptomic analysis was conducted by microarray and metabolomic analysis was conducted by GC–MS. Integrative omics was performed using MetaboAnalyst.

**Results:**

326 mRNAs showed significantly altered expression in HBE p53-/- cells post-radiation, of which 269 were upregulated and 57 were downregulated. A total of 147 metabolites were altered, including 45 that increased and 102 that decreased. By integrated analysis of both omic data, we found that in response to radiation insult, nitrogen metabolism, glutathione metabolism, arachidonic acid metabolism, and glycolysis or gluconeogenesis may be dysregulated due to p53.

**Conclusions:**

Our study provided a pilot comprehensive view of the metabolism regulated by p53 in response to radiation exposure. Detailed evaluation of these important p53-regulated metabolic pathways, including their roles in the response to radiation of cells, is essential to elucidate the molecular mechanisms of radiation-induced damage.

## Introduction

Ionizing radiation (IR) is a term applied to any type of electromagnetic radiation, such as X-rays and γ-rays, or particulate radiation, such as neutrons or alpha particles, containing sufficient energy to ionize atoms or molecules [1, 2]. The effects of radiation on cells can be characterized as a double-edged sword [3]. On one hand, IR has a therapeutic anticancer effect, acting to prevent cancer cell proliferation by inducing DNA double-strand break (DSB) damage; on the other hand, normal cells can be injured by IR [4]. For instance, a large radiation dose received over a short period of time will trigger the physiological response known as acute radiation syndrome (ARS) [5]. Furthermore, even low-dose IR exposure from diagnostic medical examinations, such as computed tomography (CT) scans, increases the overall cancer incidence by a factor of 1.54 (95% CI, 1.45–1.63; *p *< 0.001) compared to the non-exposed population [6]. Previous evidence has indicated that the hematopoietic [7], gastrointestinal [8] and vascular systems [9] are most susceptible to IR-induced injury. In our laboratory, we noted that, after the initial treatment of cancer patients with 2 Gy radiation, multiple mRNAs changed significantly compared to their levels prior to radiation treatment, with most of the affected mRNAs, such as ALAS2 and HBG2, showing the effects of hematological toxicity [10]. In fact, while radiation injury generally depends on the dose, exposure duration, and type of radiation, as well as on the gender, age and health status of the exposed individual, the underlying molecular mechanisms are related to lipid, protein and DNA damage caused by direct ionization of target molecules, and to the effects of hydroxyl radicals formed via radiolysis of water molecules [11]. Regarding the cellular response to IR insult, DSB is the most common effect, resulting in either cell death or survival with mutations that may be repaired through non-homologous end-joining (NHEJ) and homologous recombination (HR) [12, 13].

The cancer suppressor p53 was first reported in 1981 [14], and defects in p53 are directly or indirectly associated with more than 50% of all human cancers, according to the International Cancer Genome Consortium (ICGC) [15]. The location of human p53 is on the short arm of chromosome 17 (17p13), and the protein size is 393 amino acids (~ 43 kDa). p53 contains a few structural domains, including the N-terminal domain, proline-rich domain, DNA-binding region, oligomerization domain and C-terminal domain. These domains can be visualized online (https://www.ncbi.nlm.nih.gov/gene/2768677). Moreover, p53 is one of the most well-studied proteins in relation to radiation-induced DNA damage repair [16]. In the cellular response to IR, post-transcriptional modification of p53 at multiple sites triggers activation of biological functions by p53, including regulation of DNA damage repair, transient cell cycle arrest, apoptosis and terminal growth arrest [17, 18]. p53 executes these biological functions to repair IR-induced DNA damage through direct protein–protein interactions, such as the interaction with ATM, another key mediator of DSB repair [19], or indirectly through regulating the transcription of numerous p53-responsive genes [20, 21]. In recent years, increasing evidence has indicated that the accumulation of DNA damage is intimately linked to the onset of metabolic diseases, such as diabetes and obesity, and that the DNA damage response is involved in the regulation of metabolic homeostasis [22]. As the key regulator of DNA damage repair, p53 has been suggested to have an impact on systemic metabolism. Likewise, normal p53 activity is required for the physiological regulation of glucose metabolism. Yokoyama et al. indicated that upregulation of endothelial p53 led to metabolic abnormalities [23]. Meanwhile, Franklin et al. reported that p53 promotes nucleotide biosynthesis in response to DNA damage by repressing expression of the rate-limiting enzyme for glycolysis [24]. Hence, DNA damage-induced p53 activation plays critical roles in metabolic diseases, such as obesity and diabetes, suggesting that fine-tuning of the DNA damage response is essential for preventing and treating metabolic diseases [22]. Clarifying the regulatory role of p53 in metabolism would provide new insights into IR-induced DNA damage repair, radiotherapy-related resistance and DNA damage-induced dysregulation of metabolism, and thus promote more precise cancer therapy through identification of preventative and therapeutic targets for clinical application.

With the continual development of scientific technologies, transcriptomic and metabolomic analyses have been increasingly applied for uncovering molecular mechanisms and novel biomarkers based on high-throughput data [25, 26]. Transcriptomics refers to research on the complete set of RNA transcripts produced by the genome under specific circumstances, and is carried out using high-throughput methods including microarray analysis [27, 28]. Metabolomics is defined as analysis of the variations in endogenous metabolites in response to stressors such as radiation exposure in biological systems, including cells and tissues [29]. Metabolomic methods have been applied not only to discover biomarkers, but also to improve therapeutic outcomes through identification of metabolites and analysis of the variations therein [30]. We hypothesized that p53-dependent radiation injury is related to alterations of both the transcriptome and metabolome. To explore the interactions of radiation-induced transcripts with the cellular metabolism regulated by p53, we performed integrated transcriptomic and metabolomic analysis of the p53 knockout human bronchial epithelial (HBE) cell line (HBE p53-/-). Our data show that multiple mRNAs and metabolites are significantly altered after IR exposure in HBE p53-/- cells compared to HBE p53-wildtype (HBE p53-wt) cells. Moreover, “joint pathway analysis” showed that arachidonic acid metabolism, nitrogen metabolism, glutathione metabolism, glycolysis and gluconeogenesis may be controlled by p53 during the response to IR-induced damage.

## Methods and materials

### Cell line, CRISPR/cas9 method

The HBE cell line, purchased from the American Type Culture Collection, was cultured in bronchial epithelial cell growth medium containing all SingleQuots supplements (Cambrex Corporation, East Rutherford, NJ, USA) except hydrocortisone. The cells were incubated at 37°C in 5% carbon dioxide, passaged at 90% confluence, and cultured in 6- or 12-well plates. HBE cells were sent to Syngentech Ltd. (Beijing, China) for p53 knockout using the CRISPR/cas9 method. In brief, the main steps for p53 knockout using CRISPR/cas9 were as follows. First, three sgRNAs were designed: pHS-ACR-LW601(5′-CGGACGATATTGAACAATGG-3′), pHS-ACR-LW602(5′-ACCAGCAGCTCCTACACCGG-3′), and pHS-ACR-LW603(5′-GTGCTGTGACTGCTTGTAGA-3′). These three primers were inserted into the sgRNA backbone to construct the lentivirus plasmids. Then, the lentivirus plasmids were transfected with HEK 293FT cells using EpFect ™ Transfection Reagent (Syngentech). After 48 h, the liquid containing transfected lentivirus plasmids was collected for determination of viral titer. Sequence detection was used to identify the plasmids. We used the packaged p53 gene-silencing lentivirus to infect the target cell line with the optimal multiplicity of infection (MOI) value, and obtained the p53 knockout HBE cell line through Puro resistance screening.

### Transcriptome detection through microarray analysis and validation with quantitative real-time polymerase chain reaction (qRT-PCR)

HBE cells in two groups, the HBE p53-wt group and HEB p53-/- group, were harvested 24 h after exposure to 4 Gy radiation and sent to OE Biotech Ltd. (Shanghai, China) for microarray transcriptome analysis. Total RNA was extracted using TRIzol (Invitrogen, Carlsbad, CA, USA) and reverse-transcribed into cDNA using the ReverTra Ace qPCR RT Master Mix with gDNA Remover (Toyobo, Osaka, Japan) according to the manufacturers’ instructions. The GeneChip Fluidics Station 450, GeneChip Hybridization Oven 645, and GeneChip Scanner 3000 7G (Affymetrix, Santa Clara, CA, USA) were used to detect mRNA changes in the HBE p53-wt and HBE p53-/- groups after exposure to 4 Gy radiation. In our laboratory, we previously conducted integrated analysis of lncRNA-mRNA co-expression networks in α lung bronchial epithelial cells with -particle-induced carcinogenesis [31]. Fold changes greater than 2 and *p*-values less than 0.05 were considered to represent significant changes in mRNA levels. qRT-PCR was carried out in our laboratory using the Super Real PreMix Plus (SYBR Green) kit (Tiangen Biotech, Beijing, China) on the CFX96TM Real-Time system (Bio-Rad Laboratories, Hercules, CA, USA), to validate the changes and trends observed in the microarray analysis. The comparative Ct (2^−ΔΔCt^) method was used to calculate relative fold differences in mRNA expression between HBE p53-/- and HBE p53-wt cells [32, 33]. mRNA expression levels were compared using the two-tailed Student’s t-test. Differences were considered significant at *p *< 0.01 after false discovery rate correction for multiple tests.

Gene Ontology (GO) analysis of the changes in mRNA was performed, focusing on molecular functions, cellular components and biological processes (http://geneontology.org/). Kyoto Encyclopedia of Genes and Genomes (KEGG) analysis was applied to the collection of manually drawn pathway maps representing known molecular interactions, reactions and relationships for metabolism, genetic information processing, environmental information processing, cellular processes, organismal systems, human diseases and drug development (https://www.kegg.jp/kegg/pathway.html).

### Western blot analysis

Protein extraction and western blot assays were conducted according to the manufacturers’ instructions, as described previously [34, 35]. Antibodies against p53 were purchased from Cell Signaling Technology, Inc. (Danvers, MA, USA) and Abcam (Cambridge, UK). Images were captured and assessed using the ChemiDoc XRS + system (Bio-Rad Laboratories). Protein expression was quantified using Image Lab software (Bio-Rad Laboratories). At least three independent replicates were analyzed for each sample.

### Metabolomic analysis via gas chromatography-mass spectrometry (GC–MS)

Metabolomic analysis involved the extraction of metabolites and GC–MS analysis. Briefly, HBE cells were divided into HBE p53-wt and HBE p53-/- groups. The cells were harvested 24 h after exposure to 4 Gy radiation. Metabolites were extracted according to the following steps: addition of 80 μL BSTFA (including 1% TMCs) as the derivative reagent and 20 μl n-hexane, followed by addition of 11 internal standards (C8/C9/C10/C12/C14/C16, 0.8 mg/ml; C18/C20/C22/C24/C26, 0.4 mg/ml, all in chloroform) at 10 μL each, 2-min vortex mixing, and a final reaction at 70 °C for 60 min. These samples were used for the GC–MS analysis. The chromatographic conditions were as follows: Db-5 ms capillary column (30 m × 0.25 mm × 0.25 μm; J&W Scientific, Folsom, CA, USA), carrier gas of high-purity helium (purity of at least 99.999%), flow rate of 1.0 ml/min, and sample inlet temperature of 260 °C. The injection volume was 1 μL, and the solvent was added after 5 min. The temperature program was as follows: initial column chamber temperature of 60 °C, rising to 125 °C at a rate of 8 °C/min and then to 210 °C at 5 °C/min, followed by heating to 270 °C at 10 °C/min and maintenance at 305 °C for 5 min after increasing the rate of increase to 20 °C/min. The mass spectrometry conditions were as follows: electron-bombardment ion source, ion source temperature of 230 °C, quadrupole temperature of 150 °C, and electron energy of 70 eV. The scanning mode used was full scan mode (SCAN), with a scanning range of m/z 50–500.

### Bioinformatic analysis

The bioinformatic analysis involved identification of significantly altered mRNAs and metabolites, 24 h after exposure to 4 Gy radiation through a combination of multi- and single-dimension analyses. To illustrate the relationships among samples, and their metabolite expression differences, hierarchical clustering was performed and variable importance in projection (VIP) values were calculated. Pearson correlation analysis was used to detect relationships among significantly altered metabolites according to changes in biological status. Metabolic pathway enrichment data were obtained through KEGG analysis of various metabolites (http://www.kegg.jp/) [36]. A *p*-value < 0.05 was considered significant; –log *p*-values were also calculated [37].

Finally, integrated transcriptomic and metabolomic analyses were conducted using th MetaboAnalyst (https://www.metaboanalyst.ca/MetaboAnalyst/home.xhtml). The joint pathway analysis module was selected. mRNA official gene symbols and KEGG IDs of metabolites were uploaded for topology analysis, to assess the potential importance of individual molecules (i.e., nodes) based on their positions within the network. The network metrics of degree centrality and betweenness centrality were used to determine the number of links that connect to a node, and the number of shortest paths from all nodes to all other nodes that pass through a given node, respectively. Closeness centrality was used to measure the overall distance from a given node to all other nodes [38–40].

### Statistics

Statistical analysis was performed using SPSS 22.0 (IBM Corp., Armonk, NY, USA), GraphPad Prism 8.0 (GraphPad Software Inc., San Diego, USA) and R 3.2.5 (Development Core Team, Vienna, Austria) software. Data are generally provided as means ± standard deviations or counts (percentages). Differences between two groups were determined using the paired *t*-test, Wilcoxon signed rank sum test or *t*-test. A two-tailed *p*-value < 0.05 was considered statistically significant.

## Results

### Loss of p53 significantly alters mRNA expression

**Fig. 1:**
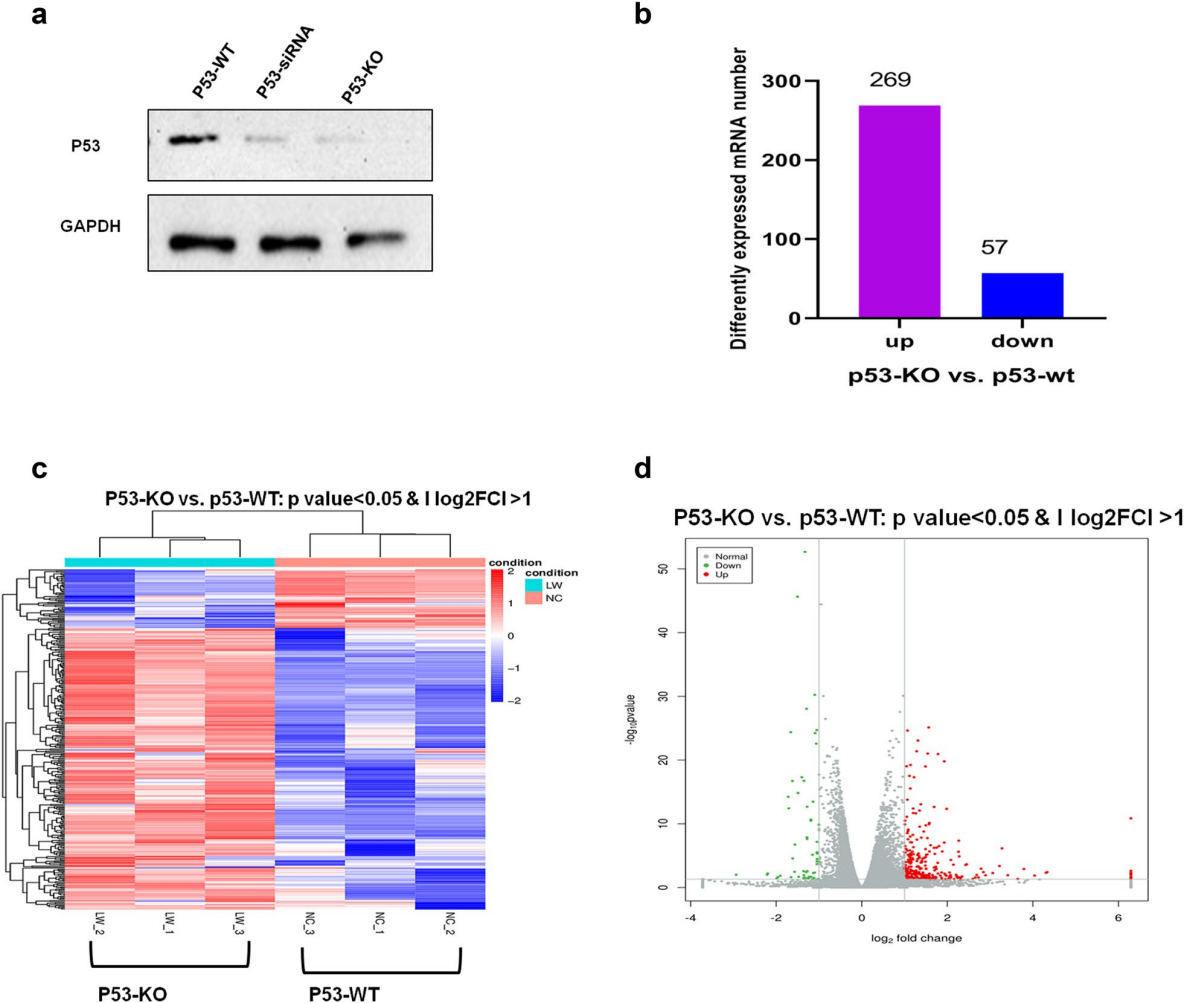
Bioinformatic analysis of the mRNA expression alteration based on HBE cells with or without CRISPR/Cas9-mediated *p53*-knockout. **a** p53 knockout efficiency was determined using western blotting. **b** Quantitative measurement of differential expressed mRNA in HBE cells with (p53-/-) or without CRISPR/Cas9-mediated *p53*-knockout (p53-wt) after 4 Gy IR. **c**. A heatmap was illustrated to show the differential expressed mRNA in HBE cells with (p53-/-) or without CRISPR/Cas9-mediated *p53*-knockout (p53-wt) after 4 Gy IR. Pink represents upregulated mRNA, and purple represents down-regulated mRNA. **d** A volcano map was illustrated to show the differential expressed mRNA in HBE cells with (p53-/-) or without CRISPR/Cas9-mediated *p53*-knockout (p53-wt) after 4 Gy IR. Red represents upregulated mRNA, green represents down-regulated mRNA and grey represents the mRNA without expression change

Western blotting was used to determine the p53 knockout efficiency (Fig. 1a) in CRISPR/Cas9-mediated *p53*-knockout HBE cells (p53-/-). To ascertain whether p53 deficiency affected mRNA expression post-radiation, we compared mRNA expression levels between the p53-/- HBE and p53-wt HBE cells. We conducted this analysis 24 h after 4 Gy γ radiation exposure, because human lung branchial epithelial cells exhibit 4 Gy-induced radiation injury (i.e., significant DNA damage) at this timepoint, based on our previous studies [31, 33, 41–43]. According to the transcriptomic data, 326 mRNAs showed significantly altered expression in HBE p53-/- cells post-radiation, of which 269 were upregulated and 57 were downregulated (Fig. 1b). Table 1 lists the top 20 differentially expressed mRNAs in p53-wt compared to p53-KO HBE cells after 4 Gy radiation. DEFA6, SLC3A1, and SCN1A were the three most strongly upregulated mRNAs, with fold changes of approximately 78, 20 and 19, respectively. C8orf86, LOC112268013, and MATK were the three most strongly downregulated mRNAs, with fold changes of 0.13, 0.216 and 0.217, respectively. The data show that p53 can regulate numerous mRNAs, indicating the critical role of p53 in the response to radiation. However, the affected mRNAs could be either directly or indirectly regulated by p53, as p53 is a transcriptional factor that may regulate other transcriptional factors in response to radiation.

**Table 1:** Top 20 differential expressed genes/mRNAs in the p53-wt vs. p53-KO HBE cells post 4 Gy radiation

Upregulated mRNA				Downregulated mRNA			
Gene-ID	Fold change	log2 (fold)	*p* value	Gene-ID	Fold change	log2 (fold)	*p* value
DEFA6	78.33913	6.291661	1.38E−11	C8orf86	0.130736	− 2.93527	0.009803
SLC3A1	20.16332	4.333661	0.003823	LOC112268013	0.216001	− 2.21089	0.00971
SCN1A	19.79819	4.307297	0.004904	MATK	0.217973	− 2.19778	0.006171
ENTHD1	16.45192	4.040184	0.013362	TMEM56-RWDD3	0.252377	− 1.98635	0.039409
RSPH6A	13.827	3.789416	0.001236	SCT	0.256374	− 1.96368	0.023869
CHRNA6	12.5158	3.645679	0.045863	P2RY4	0.267326	− 1.90333	0.014581
SLC25A6	10.65712	3.413746	0.011851	LCN2	0.303939	− 1.71815	5.85E−15
FAM163B	9.699409	3.277897	7.21E−07	SPRR2D	0.306724	− 1.70499	3.68E−13
TAS1R3	9.304103	3.217867	0.000423	SLC26A4	0.316586	− 1.65933	4.28E−25
RXFP4	8.349756	3.061734	0.005335	POTEI	0.318255	− 1.65174	0.02425
MANSC4	7.434827	2.894299	0.011991	STK32A	0.325094	− 1.62107	1.93E−17
GPR84	7.172444	2.842465	0.003795	GPR39	0.326626	− 1.61429	2.71E − 05
U2AF1	6.906392	2.787932	0.00111	CD69	0.336596	− 1.57091	0.003493
FAM19A3	6.628252	2.728628	0.026354	LPAR6	0.338796	− 1.56151	1.84E −07
CES5A	6.442318	2.68758	0.010618	NT5E	0.353169	− 1.50157	2.36E−46
KCNJ2	6.295374	2.654292	0.026678	FLNC	0.355741	− 1.4911	1.45E−15
SNCB	6.047667	2.596379	0.044324	INSC	0.360171	− 1.47325	0.021098
MAP2K6	5.47041	2.451649	0.000226	GZMB	0.378744	− 1.40071	4.97E−18
LHFPL1	5.383789	2.428622	0.000288	LOC102724219	0.389122	− 1.3617	0.032583
DEFA6	78.33913	6.291661	1.38E−11	LGALS7B	0.389833	− 1.35907	0.002712

*p* < 0.05 represents significantly changed

**Fig. 2:**
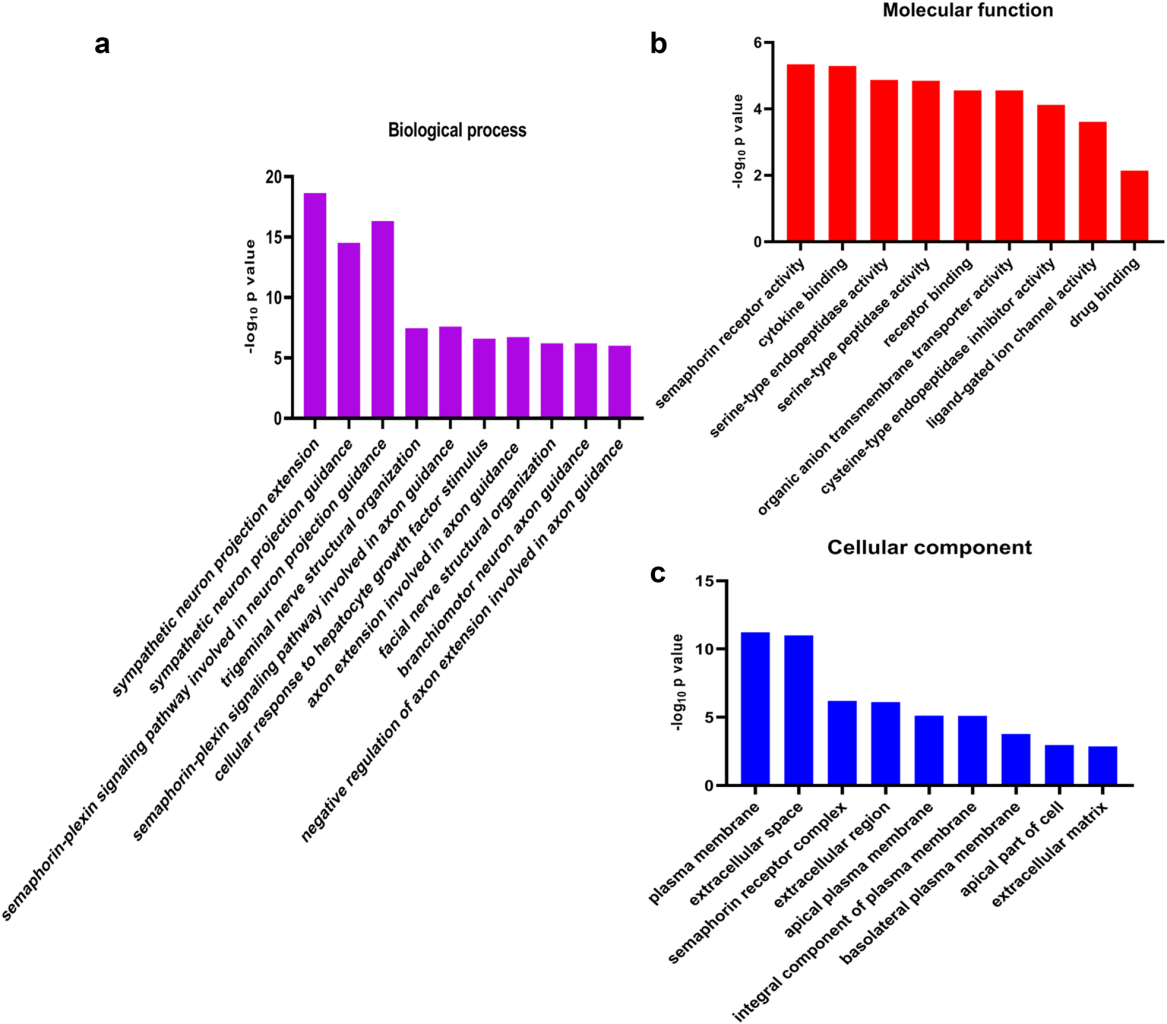
GO analysis of significantly differential expressed mRNA based on HBE cells with or without CRISPR/Cas9-mediated *p53*-knockout. **a** Enrichment of mRNA significantly differential expressed in the biological process. **b** Enrichment of mRNA significantly differential expressed in the molecular function. **c** Enrichment of mRNA significantly differential expressed in the cellular component

**Fig. 3:**
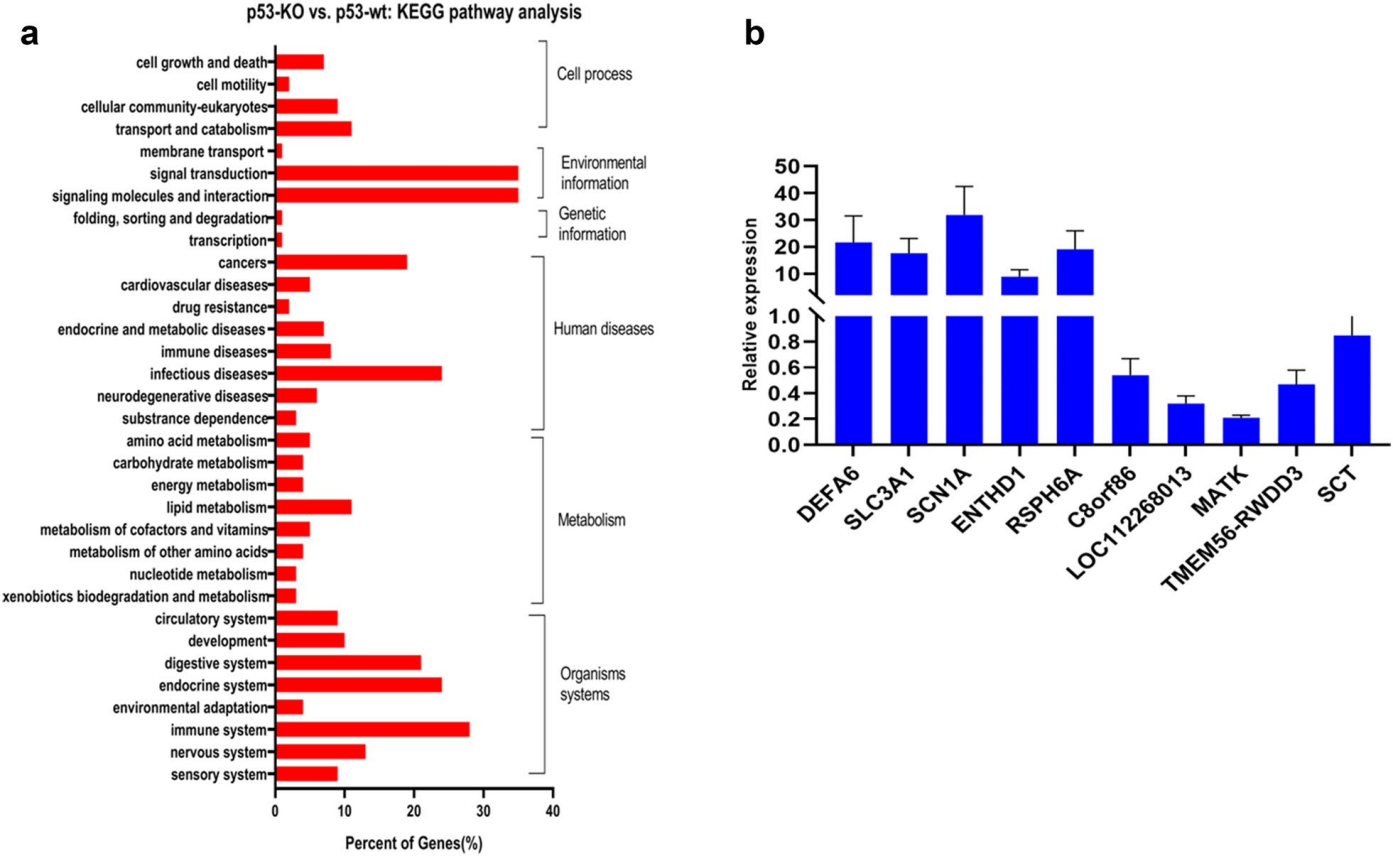
Verification of selected mRNA. **a** KEGG analysis of significantly differential expressed mRNA in HBE cells with (p53-/-) or without CRISPR/Cas9-mediated *p53*-knockout (p53-wt) after 4 Gy IR. **b** five upregualted mRNA and five downregulated mRNA were selected to verify whether the expression trend consistence with microarray assay by qRT-PCR on HBE cells with CRISPR/Cas9-mediated *p53*-knockout

Next, we constructed a heatmap of dysregulated (i.e., upregulated or downregulated) mRNAs within the HBE p53-wt and HBE p53-/- groups (Fig. 1c). Figure 1d shows a volcano plot of the two groups: the number of upregulated mRNAs was greater than the number of downregulated mRNAs. GO analysis of the 30 mRNAs showing the largest changes was conducted to identify their possible biological functions; as shown in Fig. 2, those mRNAs are mainly enriched in neuron projection extension, neuron projection guidance, and the semaphorin–plexin signaling pathway involved in neuron projection guidance. Regarding molecular functions, the top 30 mRNAs were enriched primarily in semaphorin receptor activity, cytokine binding and serine-type endopeptidase activity. Regarding cellular components, these mRNAs are mainly related to the plasma membrane, extracellular space and semaphorin receptor complex. We then conducted KEGG prediction analysis (Fig. 3a), which revealed that the signal transduction, signaling molecules and interaction, and immune system pathways are the top 3 pathways predicted in HBE p53-/- cells post-radiation, with 30–40 mRNAs enriched in each, consistent with the results of GO analysis.

We subjected the five mRNAs showing the largest changes to qRT-PCR analysis, to validate the microarray data. As shown in Fig. 3b, the expression levels of DEFA6, SLC3A1, SCN1A, ENTHD1 and RSPH6A were upregulated after 4 Gy radiation exposure, while those of C8orf86, LOC112268013, MATk, TMEM56-RWDD3 and SCT were downregulated, consistent with the microarray results.

### Loss of p53 significantly changes metabolites

To explore the p53-dependent changes in cellular metabolites after radiation insult, HBE cells were divided into HBE p53-wt and HBE p53-/- groups, as described previously. Cells were harvested 24 h after 4 Gy radiation exposure, and their metabolite profiles were constructed based on the GC–MS data. A total of 35 metabolites changed significantly in HBE p53-/- cells compared to p53-wt cells (*p *< 0.05). A total of 147 metabolites were altered, including 45 that increased and 102 that decreased. Table 2 lists the top 35 most altered metabolites, including 5 that increased and 30 that decreased. Among them, erythronic acid lactone, hydroxylamine, oleamide, 1,3-diphosphoglycerol and O-phosphoethanolamine were upregulated, while 4-hydroxymandelic acid, erythronic acid, epsilon-caprolactam, allothreonine and metharbital were downregulated, in HBE p53-/- cells post-radiation. The dysregulated metabolites belong to multiple metabolic classes, including lactones, phenols, organo-oxygen compounds, carboxylic acids (and their derivatives purine nucleosides, and fatty acyls), indicating that p53 deficiency leads to dysregulation of multiple metabolic classes, which may result in changes in related metabolic pathways. The data show that p53 plays a critical role in regulating radiation-induced metabolic changes. To identify the metabolites most closely associated with the p53 deficiency, VIP analysis was performed and the results are shown in Table 2. Using 1.00 as the VIP cutoff score, 35 metabolites were identified as being potentially closely associated with p53 deficiency.

**Table 2:** 35 differential expressed metabolites in p53-wt vs.p53-KO HBE cells post 4 Gy radiation

Metabolites	VIP*	log2 (FC)	*p*-value	Class	Sub class
Erythronic acid lactone	1.06922	0.6158	6.9E−05	Lactones	Organoheterocyclic compounds
Hydroxylamine	1.34941	0.19798	0.04045	Homogeneous other non-metal compounds	Homogeneous non-metal compounds
4-hydroxymandelic acid	1.36889	− 0.6996	0.00142	Phenols	Benzenoids
Erythronic acid	1.42277	− 1.7093	0.00929	Organooxygen compounds	Organic oxygen compounds
Epsilon-caprolactam	1.34602	− 0.7127	0.00061	Lactams	Organoheterocyclic compounds
Allothreonine	1.46924	− 0.5049	0.00792	Carboxylic acids and derivatives	Organic acids and derivatives
Metharbital	1.41033	− 1.0301	0.00381	Diazines	Organoheterocyclic compounds
Nicotinic acid	1.42734	− 0.5283	0.00083	Pyridines and derivatives	Organoheterocyclic compounds
3,3-dimethyl-1-hydroxy-cyclohexene	1.009	− 1.3095	0.00151	–	–
Cyanoalanine	1.40793	− 0.7455	9.4E−05	–	–
2,4-diaminobutyric acid	1.4873	− 0.793	0.03173	Carboxylic acids and derivatives	Organic acids and derivatives
Isocitric acid	1.43121	− 1.3229	0.01103	Carboxylic acids and derivatives	Organic acids and derivatives
Alpha-tocopherol	1.47328	− 1.0729	0.00435	Prenol lipids	Lipids and lipid-like molecules
Arbutin	1.06715	− 1.2865	0.00725	Organooxygen compounds	Organic oxygen compounds
5-hydroxymethyl-2-furoic acid	1.38372	− 2.0063	0.00456	Furans	Organoheterocyclic compounds
Alloxanoic acid	1.15251	− 2.265	0.0279	–	–
3-hydroxybenzoic acid	1.4286	− 1.47	0.00704	Benzene and substituted derivatives	Benzenoids
4-hydroxyphenylacetic acid	1.34886	− 0.7195	0.01778	Phenol esters	Benzenoids
Succinylacetone	1.00743	− 3.6389	0.01924	Keto acids and derivatives	Organic acids and derivatives
Chlorogenic acid	1.01029	− 2.1308	0.00706	Organooxygen compounds	Organic oxygen compounds
Tocopherol acetate	1.3993	− 1.8671	0.01908	Prenol lipids	Lipids and lipid-like molecules
Pseudo uridine	1.24524	− 0.4121	0.03491	Nucleoside and nucleotide analogues	Nucleosides, nucleotides, and analogues
Epicatechin	1.37918	− 0.9262	0.01609	Flavonoids	Phenylpropanoids and polyketides
O-phosphoethanolamine	1.37653	0.87116	0.04621	Organic phosphoric acids and derivatives	Organic acids and derivatives
Azelaic acid	1.13179	− 1.3106	0.00278	Fatty Acyls	Lipids and lipid-like molecules
Beta-glutamic acid	1.22441	− 0.4523	0.03566	–	–
Lactobionic acid	1.34112	− 1.0772	0.01633	–	–
Arsenate	1.03192	− 1.9182	0.00398	Miscellaneous mixed metal/non-metals	Mixed metal/non-metal compounds
Palatinitol	1.27221	− 0.7068	0.0263	–	–
L-allothreonine	1.38117	− 0.6869	0.04396	Purine nucleosides	Nucleosides, nucleotides, and analogues
Oleamide	1.02286	0.2853	0.01158	Fatty Acyls	Lipids and lipid-like molecules
1,3-diphosphoglycerol	1.12954	1.40969	0.02935	–	–
D-erythro-sphingosine	1.24636	− 0.5613	0.02118	Organonitrogen compounds	Organic nitrogen compounds
Phytosphingosine	1.11889	− 0.6993	0.0023	Organonitrogen compounds	Organic nitrogen compounds

A positive value indicates up regulation and a negative value indicates down regulation

* Vip variable important in projection, The larger the VIP, the greater the contribution of this variable to the grouping; log2 (FC): the ratio of the average expression amount of metabolites in the two groups of samples

**Fig. 4:**
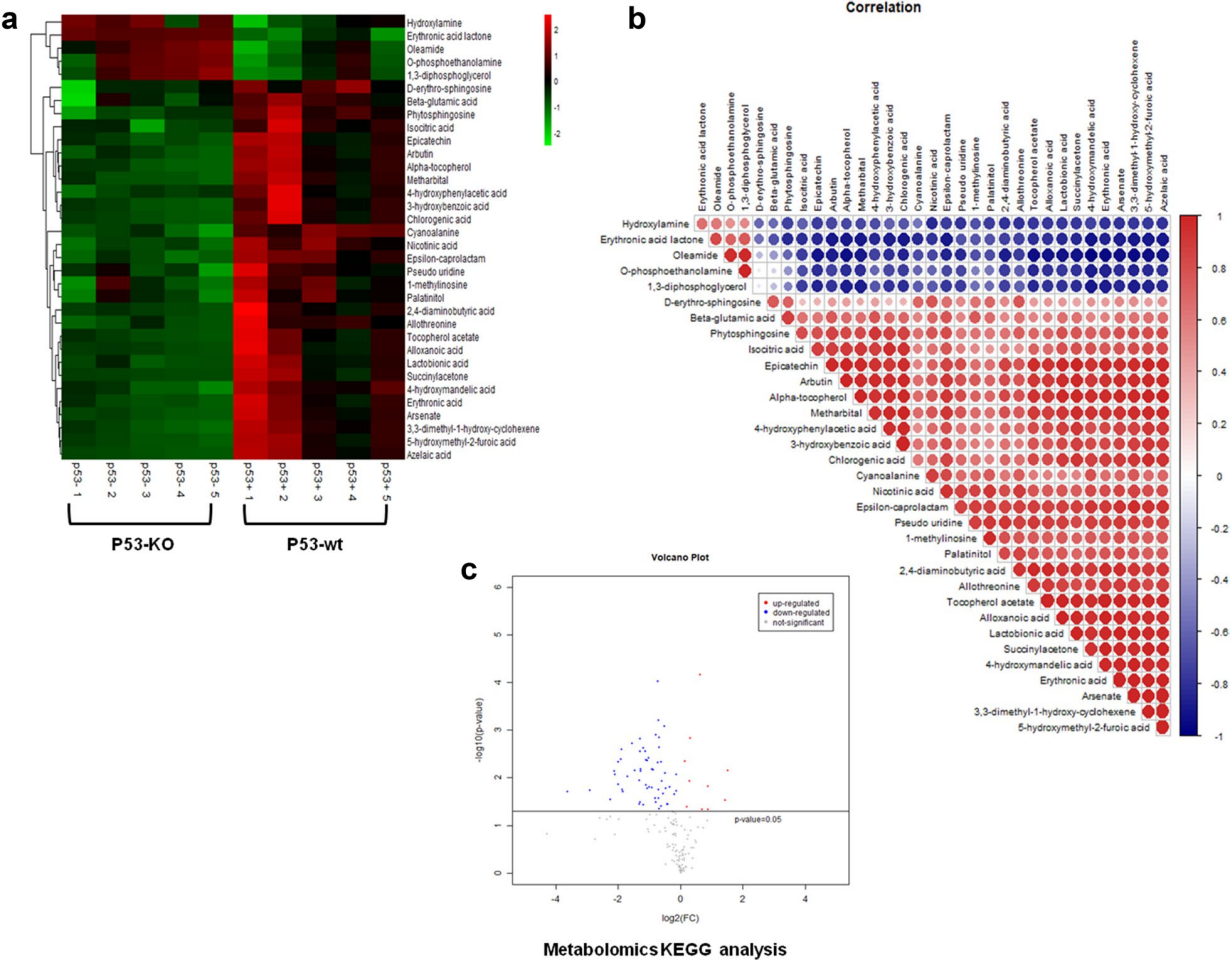
Bioinformatic analysis of metabolomics. **a** A heatmap was used to visually represent upregulated and downregulated metabolites. **b** A volcano was used to visually represent upregulated and downregulated metabolites. **c** The interactions among altered metabolites were analyzed by pearson correlation analysis

**Fig. 5:**
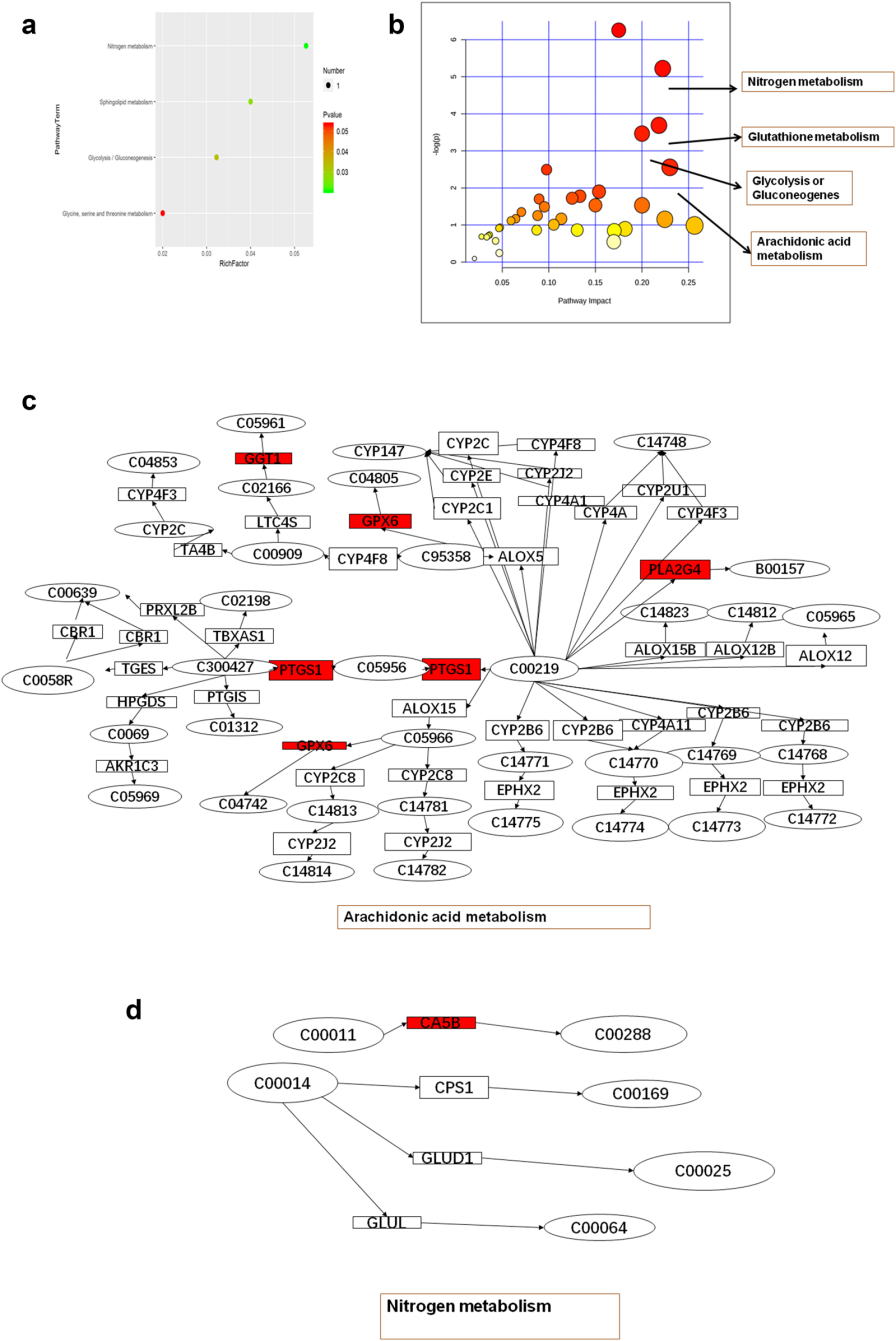
Integrated transcriptomic and metabolomic analyses of p53-dependent metabolic pathways. **a** KEGG analysis of significantly altered metabolites. **b** The mRNA enrichment and metabolic pathway analyses was based on joint pathways using MetaboAnalyst 4.0. **c** The arachidonic acid metabolism pathway with altered significantly mRNA (red) in HBE cells with CRISPR/Cas9-mediated *p53*-knockout. **d** The nitrogen metabolism pathway with altered significantly mRNA (red) in HBE cells with CRISPR/Cas9-mediated *p53*-knockout

To further understand the alterations in p53 knockout-related metabolites post-radiation, we used a heatmap and volcano plot to visually represent upregulated and downregulated metabolites. As shown in Fig. 4a, compared to the p53-wt group, metabolites were significantly downregulated in the p53 knockout group post-radiation. The volcano plot also shows that more metabolites were downregulated than upregulated (Fig. 4b). To investigate the interactions among these altered metabolites, we conducted Pearson correlation analysis. As shown in Fig. 4c, the expression level of erythronic acid lactone was positively correlated with those of hydroxylamine and oleamide, while 1,3-diphosphoglycerol expression was positively correlated with that of O-phosphoethanolamine. On the other hand, phytosphingosine expression was negatively correlated with that of erythronic acid lactone. The correlation analysis showed that metabolites may exhibit interactions during radiation-induced cell injury. The metabolomic data confirmed that p53 has a wide range of regulatory effects on intracellular metabolism in response to radiation. We also performed KEGG analysis, which indicated that the nitrogen metabolism, sphingolipid metabolism, glycolysis/gluconeogenesis and glycine, serine and threonine metabolism pathways changed significantly based on the 35 most altered metabolites (Fig. 5a). These data suggest that p53 may regulate metabolites via these metabolic pathways.

### Integrated transcriptomic and metabolomic analyses of p53-dependent metabolic pathways

**Fig. 6:**
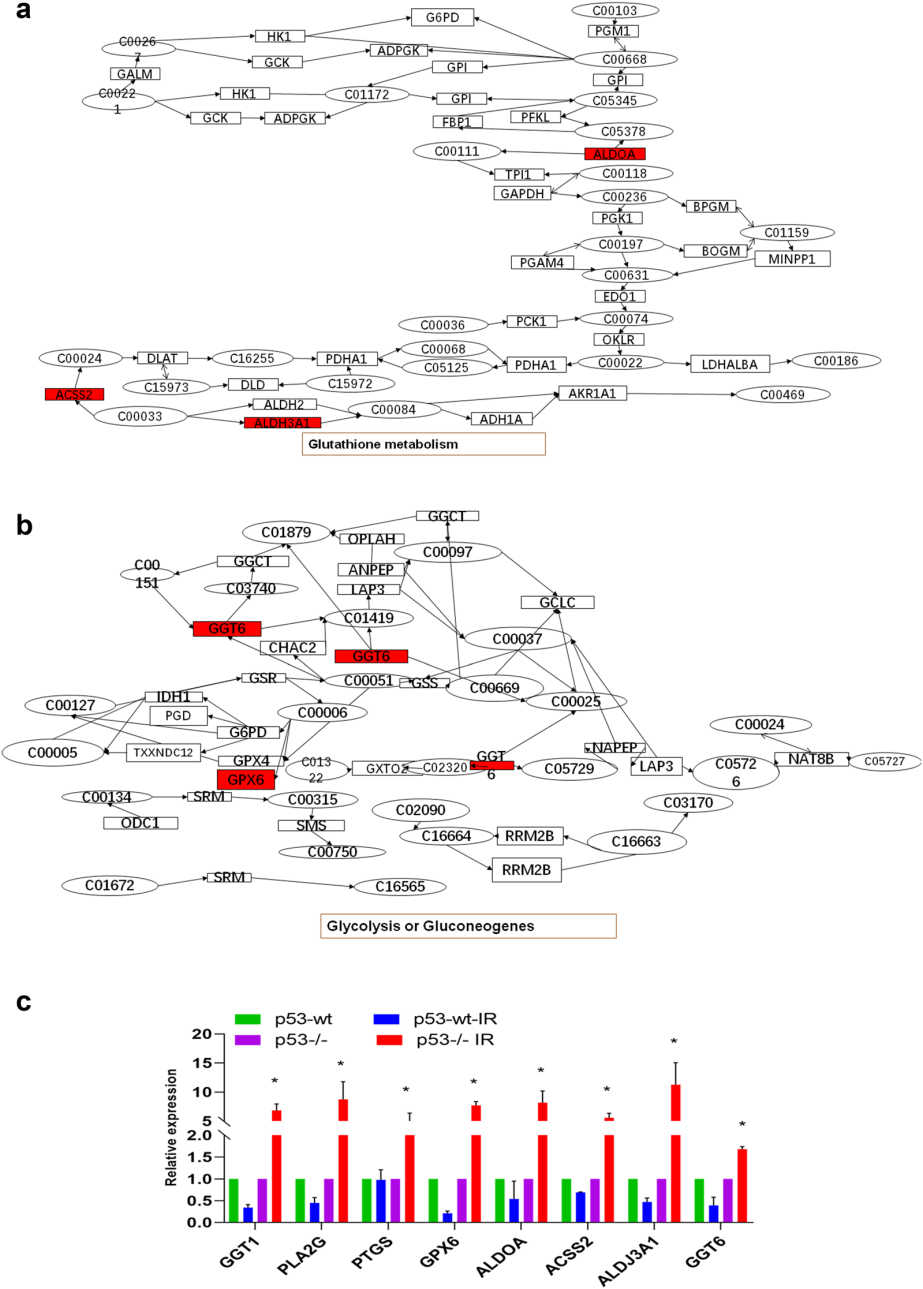
Integrated transcriptomic and metabolomic analyses of p53-dependent metabolic pathways. **a** The glutathione metabolism pathway with altered significantly mRNA (red) in HBE cells with CRISPR/Cas9-mediated *p53*-knockout. **b** The glycolysis or gluconeogenes metabolism pathway with altered significantly mRNA (red) in HBE cells with CRISPR/Cas9-mediated *p53*-knockout. **c** GGT1, PLA2G, PTGS, GPX6, ALDOA, ACSS2, ALDH3A1, GGT6 expression levels were determined using qRT-PCR in HBE cells of p53-wt, p53-wt treated with 4 Gy radiation (p53-wt –IR), p53-/-, and p53-/- treated with 4 Gy radiation (p53-/- IR), respectively. The data are presented as the means ± SDs from three independent experiments; * *p *< 0.05 between different groups

Multiple-omic analysis is a comprehensive modality that can greatly increase the validity of results [44]. Most importantly, integrating metabolic and transcriptomic data can uncover significantly perturbed pathways at both the metabolic and transcriptional levels, thereby providing a basis for further investigation of the underlying molecular mechanisms [45]. Thus, we used MetaboAnalyst 4.0 to conduct joint pathway analysis of transcriptomic and metabolomic data [40, 46]. The mRNA enrichment and metabolic pathway analyses revealed 37 significantly altered pathways, at both the metabolomic and mRNA expression levels, in HBE p53-/- cells, where these pathways are involved in nitrogen metabolism, glutathione metabolism, glycolysis or gluconeogenesis, and arachidonic acid metabolism, among other processes (Table 3). Figure 5b shows the altered pathways, including the metabolic pathways of interest. Four metabolic pathways, i.e., nitrogen metabolism, glutathione metabolism, arachidonic acid metabolism, and glycolysis or gluconeogenesis, had *p*-values < 0.05 and impact coefficients > 0.2, indicating that, in response to radiation insult, these pathways may be dysregulated due to p53. These four metabolic pathways showed significant differences in HBE p53-/- cell mRNA expression after radiation exposure (highlighted in red in Fig. 5c, b). As shown in Fig. 5c, in the arachidonic acid metabolism pathway, the expression levels of GGT1, PLA2G, PTGS and GPX6 were altered in HBE p53-/- cells post-radiation. Figure 5d shows that, in the nitrogen metabolism pathway, CA5B was altered in HBE p53-/- cells post-radiation, while Fig. 6a shows that in the glutathione metabolism pathway, ALDOA, ACSS2 and ALDH3A1 were altered in HBE p53-/- cells; finally, Fig. 6b shows that in the glycolysis or gluconeogenesis pathway, GGT6 and GPX6 were affected in HBE p53-/- cells. These data indicate that the changes seen in mRNA expression levels may be related to p53-dependent alteration of metabolic pathways (Additional file 1: Fig. S1).

**Table 3:** Integration of pathways of both the metabolites and metabolic genes

Pathway name	Match status	P	Impact
Fructose and mannose metabolism	1/40	0.37213	0.25641
Tyrosine metabolism	3/88	0.077606	0.22989
Pyrimidine metabolism	2/99	0.31535	0.22449
Nitrogen metabolism	2/10	0.0054	0.22222
Glutathione metabolism	3/56	0.024996	0.21818
Glycolysis or Gluconeogenesis	3/61	0.03124	0.2
Phenylalanine metabolism	1/21	0.21603	0.2
Pyruvate metabolism	1/45	0.40794	0.18182
Arachidonic acid metabolism	5/81	0.001919	0.175
Propanoate metabolism	1/48	0.42847	0.17021
Purine metabolism	2/166	0.57811	0.1697
Thiamine metabolism	1/14	0.14958	0.15385
Biotin metabolism	1/21	0.21603	0.15
Taurine and hypotaurine metabolism	1/16	0.1691	0.13333
Retinol metabolism	1/47	0.42171	0.13043
Linoleic acid metabolism	1/17	0.17869	0.125
Drug metabolism-cytochrome P450	2/98	0.31104	0.1134
Ether lipid metabolism	1/39	0.36472	0.10526
Nicotinate and nicotinamide metabolism	2/42	0.082504	0.097561
Alpha-Linolenic acid metabolism	1/22	0.2251	0.095238
Glycine, serine and threonine metabolism	2/68	0.18248	0.089552
Primary bile acid biosynthesis	2/92	0.28513	0.087912
Pentose phosphate pathway	1/47	0.42171	0.086957
Glycerophospholipid metabolism	2/86	0.25918	0.070588
Histidine metabolism	1/32	0.31045	0.064516
Fatty acid degradation	2/102	0.32824	0.059406
Starch and sucrose metabolism	1/43	0.39386	0.047619
Fatty acid biosynthesis	1/129	0.78338	0.046875
beta-Alanine metabolism	1/44	0.40094	0.046512
Cysteine and methionine metabolism	1/71	0.56461	0.042857
Glyoxylate and dicarboxylate metabolism	1/56	0.47992	0.036364
Sphingolipid metabolism	1/58	0.49207	0.035088
Folate biosynthesis	1/61	0.50978	0.033333
Metabolism of xenobiotics by cytochrome P450	2/145	0.50302	0.027778
Steroid hormone biosynthesis	1/199	0.90892	0.020202

To validate the transcriptomic and metabolomic results, HBE cells were divided into four groups: p53-wt, p53-wt treated with 4 Gy radiation (p53-wt –IR), p53-/-, and p53-/- treated with 4 Gy radiation (p53-/- IR). Cells were harvested 24 h after 4 Gy radiation, and GGT1, PLA2G, PTGS, GPX6, ALDOA, ACSS2, ALDH3A1, GGT6 expression levels were determined using qRT-PCR. As shown in Fig. 5g, compared to the untreated p53-wt group, the GGT1, PLA2G, PTGS, GPX6, ALDOA, ACSS2, ALDH3A1, GGT6 expression levels were significantly reduced in p53-wt after treatment with 4 Gy radiation. On the other hand, compared to untreated p53-/- cells, GGT1, PLA2G, PTGS, GPX6, ALDOA, ACSS2, ALDH3A1, GGT6 expression levels were significantly elevated in p53-/- cells after treatment with 4 Gy radiation (Fig. 6c). We also performed study on the effects of p53 knockout on the radiation-associated epithelial to mesenchymal (EMT) in HBE cells. The data is shown at Additional file 2: Fig. S2, illustrating the p53 knockout would promote development of radiation-associated epithelial to mesenchymal.

These findings are thus consistent with the transcriptomic and metabolomic results, providing further evidence that the involvement of these genes in dysregulation of radiation-related metabolic pathways may be p53-dependent, and also supporting the importance of p53 in the regulation of radiation-induced cell damage.

## Discussion

Since p53 was first identified, numerous studies have indicated that p53 plays essential roles in biological processes [47]. Our laboratory has focused on p53 regulation of radiation-induced injury over the past decade, and this study was performed to assess the potential interactions of mRNA and metabolites regulated by p53 during the cellular response to radiation. In this study, 326 mRNAs changed significantly in p53-/- cells in response to 4 Gy radiation exposure, including 269 mRNAs that increased and 57 that decreased. Furthermore, 147 metabolites changed significantly, of which 45 increased and 102 decreased. Integrated transcriptomic and metabolomic analysis indicated that GGT1, PLA2G, PTGS, GPX6, ALDOA, ACSS2, ALDH3A1, GGT6, which are involved in nitrogen metabolism, glutathione metabolism, glycolysis or gluconeogenesis, and arachidonic acid metabolism, are regulated by p53. Our study provides deep insight into p53-mediated mRNA and metabolic regulation in response to radiation insult, and identifies key mRNAs and metabolites, as potential targets of p53, for future studies of the underlying molecular mechanisms.

As a key cancer suppressor, p53 has been intensively studied in terms of its role in regulating cellular processes such as autophagy, cell cycle arrest and senescence, when cells are subjected to stress [48]. In particular, p53 plays a crucial role in the cellular response to radiation. In previous studies, the p53-mediated cellular response to radiation, which includes apoptosis and cell cycle arrest, appeared unnecessary for its cancer-suppressing function; moreover, compared to p53 knockout mice, cancer was not as easily induced in p21 or PUMA knockout mice [49, 50]. Recently, increasing evidence has shown that cancer does not occur in mice with p53 mutations, which nonetheless retain p53 activity for regulation of energy metabolism; this suggests that p53 activities in the context of metabolic regulation may be critical for suppression of tumorigenesis [51]. Numerous cancer studies of the metabolic pathways regulated by p53 have been conducted recently [52–54]. As noted by Gomes et al. in their review article, p53 activation is involved in the complex process of reprogramming cancer glucose metabolism [55]. However, no studies have used multiple-omic techniques to provide a global perspective on p53-dependent metabolic regulation of the response to radiation stress. Thus, the goal of this study was to reveal the transcriptomic and metabolomic alterations that occur in response to radiation insult.

Several studies of radiation-induced transcriptome changes have been published in recent years. Stankevicius et al. reported that the response of human colorectal carcinoma (DLD1) cells to a single exposure to 2 or 10 Gy induced differential expression of 1575 genes, and KEGG analysis showed that the p53 pathway was the most significantly altered in those cells [56]. Moreno-Villanueva et al. investigated gene expression in individual human T lymphocytes 3 h after ex vivo exposure to 2 Gy γ-rays, and identified a group of TP53-responsive genes that are involved in the interferon-γ pathway [57]. Molt-4-LXSN cells expressing wild-type p53, and p53-deficient Molt-4-E6 cells, were γ-irradiated; 14 h later, multiple genes were found to be dysregulated in p53-deficient Molt-4-E6 cells, including PMAIP1, CDKN1A and FAS, which are involved in the p53 pathway [58]. These transcriptomic studies indicate that p53 plays a key regulatory role in IR-induced damage. Our data are consistent with previous research showing that the expression levels of multiple mRNAs were significantly altered due to changes in p53 status after radiation exposure. As a cancer suppressor, p53 deficiency commonly leads to tumorigenesis. Thus, although the p53-/- HBE cell model used in this study may not be optimal in all circumstances, it could be used to investigate the p53-mediated metabolic regulation associated with radiation stress.

Integration of transcriptomic and metabolomic data may yield greater insight into radiation-induced damage than either approach alone. In their review, Volkova et al. noted that multiple-omics approaches are useful for radioecological research [59]. Moreover, Sproull et al. stated that -omics techniques to assess individuals exposed to radiation injury, such as those affected by radiological or nuclear events, are necessary [60]. In our study, the genes altered in response to radiation (GGT1, PLA2G, PTGS, GPX6, ALDOA, ACSS2, ALDH3A1, GGT6) were involved in nitrogen metabolism, glutathione metabolism, glycolysis or gluconeogenesis and arachidonic acid metabolism, and were regulated by p53. Nitrogen metabolism has been reported to regulate global gene expression in human fibroblasts in response to γ-rays, by activating the p53 signaling pathway [61]. In glutathione metabolism, glutamine provides additional carbon and nitrogen for cell growth [62], thereby driving glutathione synthesis, and contributes to radiation sensitivity in lung cancer cell lines [62]. Glycolysis and gluconeogenesis have been extremely well characterized in many studies; during glycolysis, the glucose molecule is divided into two pyruvate molecules, which leads to the generation of lactate in cancer cells. Metabolic pathways based on glycolysis include the pentose phosphate and hexosamine biosynthetic pathways [63]. p53 also plays a mediating role in glycolysis. A previous report indicated that p53 induces TIGAR (a TP53-induced glycolysis and apoptosis regulator) to decrease PFK1 (6-phosphofructokinase 1) activity and reduce the glycolytic rate [64]. Zhang et al. showed that p53 negatively regulates glycolysis under hypoxic conditions in cancer cells, through targeting of Ras-related associated with diabetes (RRAD) [65]. Arachidonic acid is metabolized to epoxyeicosatrienoic acid through the p53-mediated apoptotic pathway [66]. Although these metabolic pathways have previously been reported to show associations with p53, few studies have focused on radiation-induced damage to cellular metabolic pathways under conditions of p53 deficiency. The present study highlights the most important metabolic pathways regulated by p53 in response to radiation. Furthermore, our results indicate that exposure to radiation under p53 deficiency conditions can result in significant dysregulation of both the transcriptome and metabolome.

Despite using multiple-omics to observe global biological changes following exposure to radiation, and providing important data showing the involvement of p53-regulated mRNA and metabolites in the radiation-induced damage response, this study had limitations. First, only one cell line was used; studies using multiple cell lines could validate the results. Second, we did not investigate whether the observed transcriptomic and metabolomic alterations under p53 deficiency conditions are dose- or time-dependent. Third, we did not perform functional analyses to uncover the molecular mechanisms underlying the observed changes.

## Conclusions

Our study provided a pilot comprehensive view of the metabolism regulated by p53 in response to radiation exposure. Detailed evaluation of these important p53-regulated metabolic pathways, including their roles in the response to radiation of cells, is essential to elucidate the molecular mechanisms of radiation-induced damage.

## Supplementary information

**Additional file 1: Figure** **S1.** CRISPR/Cas9-mediated *p53*-knockout. A. sgRNA backbone was used to construct the vector. B. Sequence detection to test the p53 knockout. C. Cells were transfected with vectors.

**Additional file 2: Figure**  **S2.** Western blot assay was performed to study the effects of p53 knockout on the radiation-associated epithelial to mesenchymal (EMT) biomarkers in HBE cells.

## Data Availability

Not applicable.
