# Supplementary figures and images for: Integrated analysis of transcriptomic and metabolomic profiling reveal the p53 associated pathways underlying the response to ionizing radiation in HBE cells
Source: Cell Biosci. 2020 Apr 15;10:56. doi: 10.1186/s13578-020-00417-z (PMC7160934; doi:10.1186/s13578-020-00417-z)

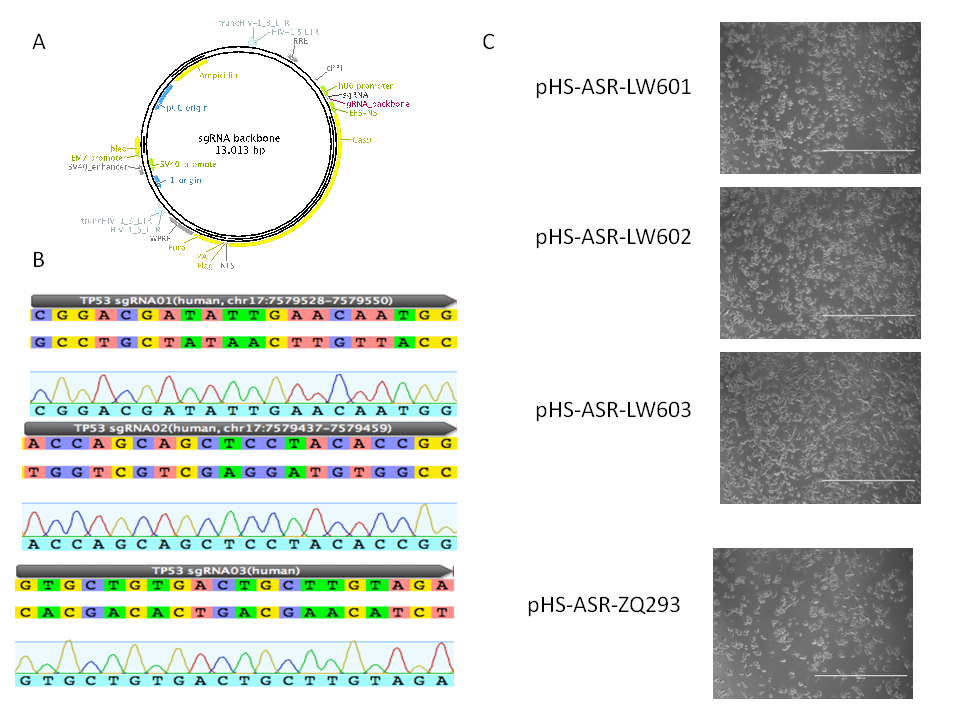

Supplement: Supplementary file 1 — Additional file 1: Figure S1. CRISPR/Cas9-mediated p53-knockout. A. sgRNA backbone was used to construct the vector. B. Sequence detection to test the p53 knockout. C. Cells were transfected with vectors. [file 13578_2020_417_MOESM1_ESM.tif]

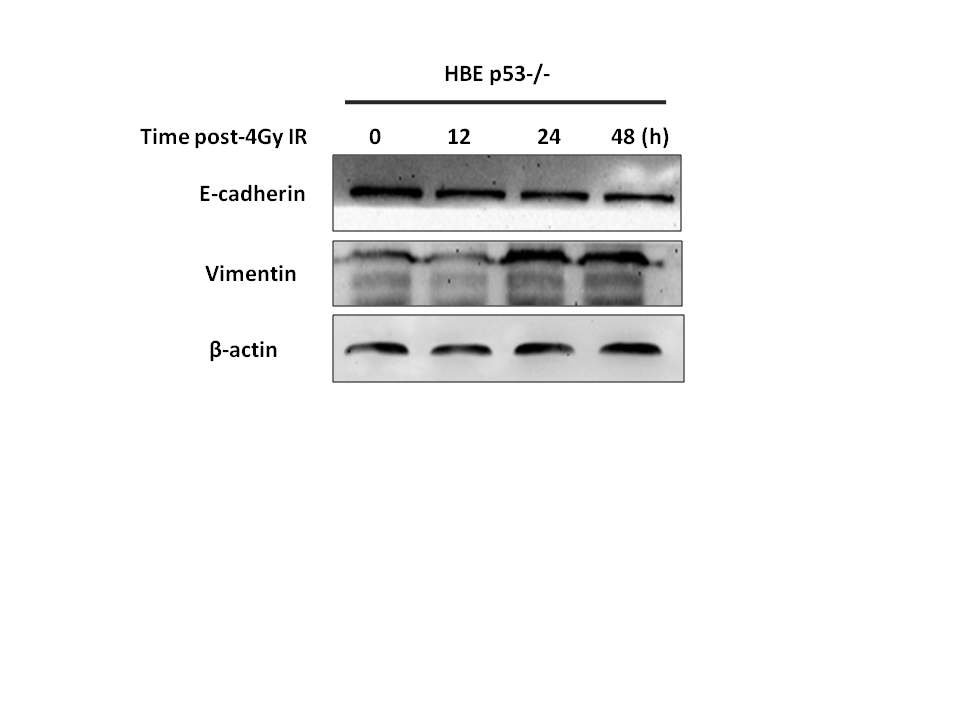

Supplement: Supplementary file 2 — Additional file 2: Figure S2. Western blot assay was performed to study the effects of p53 knockout on the radiation-associated epithelial to mesenchymal (EMT) biomarkers in HBE cells. [file 13578_2020_417_MOESM2_ESM.tif]
